# Treatment of saline municipal wastewater using hybrid growth system

**DOI:** 10.1186/s13036-016-0030-7

**Published:** 2016-08-31

**Authors:** N. Salmanikhas, M. Tizghadam, A. Rashidi Mehrabadi

**Affiliations:** Department of Water and Environmental Engineering, Abbaspour School of Engineering, ShahidBeheshti University, Tehran, Iran

**Keywords:** Saline municipal wastewater, Hybrid growth, Activated sludge

## Abstract

**Background:**

In this study, a hybrid treatment system (Fluidized Bed positioned in a biological reactor of an Activated Sludge process) was used to treat saline domestic wastewater. The performance of the mentioned hybrid system was compared with the conventional activated sludge. A pilot study was conducted, and Chemical Oxygen Demand (COD), Electrical Conductivity (EC), Total Dissolved Solids (TDS) and pH were measured to investigate treatment efficiency. Three saline wastewater samples with salt concentrations of 0.5, 1, and 1.5 % and detention times of 2, 4 and 6 h were loaded into both rectors of hybrid system and activated sludge.

**Results:**

The results showed that Chemical Oxygen Demand (COD) removals at salt concentrations of 0.5, 1, 1.5 % were equal to 80, 71, 48.5 for the hybrid system and 62, 47.7, 26.5 for the activated sludge system respectively. Likewise, similar results obtained for other contamination indices indicating the superiority of the hybrid system in comparison to activated sludge system. Moreover, another advantage of the hybrid system was that the activated sludge needed sludge returning while sludge returning was not required in the hybrid system. In addition, by loading fixed rate of air into both systems, dissolved oxygen concentration in the hybrid reactor is higher than the conventional reactor.

**Conclusions:**

Therefore, the hybrid system had a significantly higher efficiency than conventional reactor to treat saline domestic wastewater.

## Background

In coastal cities, the quality of wastewaters entering the treatment plant is affected by the high levels of groundwater and leakage of waters into the collection system. The salinity of groundwater in these regions is relatively high [23]. Discharging saline wastewaters without proper treatment, significantly influences the life of aquatics causing the migration, death, and devastation of organisms and imbalance of the ecosystem, plus negatively affecting water quality for drinking and agricultural use [6, 29]. Thus, there are strict rules which have been set for mandatory treatment of such wastewaters as removal of organic compounds and salts in most countries [2]. To treat saline wastewaters, using physical and chemical treatment processes are common, while biological treatment has been underused due to the high concentration of salinity (mainly NaCl) [12, 15, 24]. On the other hand, regular physical and chemical treatment processes of saline wastewaters like flocculation and coagulation, ion exchange and softening saline wastewaters, ultrafiltration (UF), the combination of centrifugation (CF) and ultrafiltration, reverse osmosis (RO), and electro dialysis (ED) are noticeably expensive. Therefore, using these methods are limited in the majority of circumstances [9, 15, 16, 31]. Recently, studies have mainly focused on the application of proper, cheap and environmentally-friendly biological treatment systems because of the easier maintenance of biological methods, and the relatively low administrative and operational costs of them [8, 10].

The effectiveness of biological methods in nutrients and organic compounds removal depends on the adaptability of microorganisms in saline condition and their sustainability. Hence, some microorganisms can regulate their intracellular conditions when confrontation to environmental challenges occurs. Similarly, their growth kinetics and decomposition rates are also changes [14]. Therefore, in the biological treatment of saline wastewaters, it is necessary to proceed towards further adaptation of microorganisms to the environment like highly saline wastewaters [1, 20, 28].

In saline wastewaters, one of important parameters was Total Dissolved Solids (TDS) or salinity affecting the osmotic force and ionic compound [22]. These two physical factors significantly influence the ability of microorganisms for survival and proliferation. While in wastewaters with high TDS levels (salt concentration above 1 %), microorganisms experience diminished cytoplasmic activity, eventually causing sudden plasmolysis [14, 32].

Another problem in saline wastewaters treatment is referred to their high concentration of suspended solids or turbidity in their effluent. The reason is that the salt present in the wastewater increases buoyancy force and consequently decreases sedimentation [14]. In other words, the difference of density between the environment and water is low in saline wastewaters, and thus, sedimentation of bacteria are slow and, so it is hard to retain the bacteria in the system [4, 30].

In this regard, the aim of this study is to use a fluidized bed, positioned in a biological reactor of an activated sludge process for the first time, in order to improve bacteria growth condition to synchronize suspended and attached growth, which leads to increase sustainability and adaptability of the relevant microorganisms.

## Methods

### Operational conditions

The operational conditions of this study are showed in Table 1. The operational temperature during the study was 30 ± 2 °C. This study was conducted continuously over 180 days, nine 20-day periods.

**Table 1:** Operational conditions for hybrid and conventional systems in saline wastewater treatment

Days	TDS (mg/l)	HRT (h)	% Qin	COD (mg/l)	BOD_5_ (mg/l)	NH^+^_4_ (mg/l)	TP (mg/l)	pH	EC (μs/cm)	Cl^−^(mg/l)
1–20	0.5	2	25	414 ± 40	242.6 ± 3.7	27.3 ± 5	11.8 ± 1.7	8 ± 0.5	9030 ± 121.2	2438.3 ± 131.7
21–40	0.5	4	50	413 ± 11.1	240 ± 11.7	25.6 ± 6.1	11.1 ± 2.4	7.9 ± 0.3	8966.6 ± 152.7	2422.3 ± 133.6
41–60	0.5	6	75	383.3 ± 7.6	253.3 ± 17.5	16.1 ± 1	10 ± 2	8 ± 0.3	9073.3 ± 125	2463.3 ± 62.5
61–80	1	2	50	396 ± 37.3	262.3 ± 37.6	20 ± 2	12.3 ± 0.6	8.3 ± 0.5	18083.3 ± 104.08	5197.3 ± 92.6
81–100	1	4	75	439.3 ± 27.6	244 ± 13.8	26 ± 3.6	8.5 ± 0.6	7.9 ± 0.5	18,000 ± 100	5257.3 ± 161.1
101–120	1	6	25	418.3 ± 55.3	230.3 ± 17.7	25 ± 5.2	13.9 ± 0.4	8.4 ± 0.3	18,350 ± 132.2	5017.3 ± 275.8
121–140	1.5	2	75	380.6 ± 17.7	247.3 ± 34.4	27.3 ± 6.0	13.2 ± 1.1	8.2 ± 0.3	27383.3 ± 104	8110 ± 252.8
141–160	1.5	4	25	408.3 ± 16.5	286.3 ± 9.8	28.8 ± 5.6	11.9 ± 0.6	8.6 ± 0.2	27233.3 ± 251.6	8289.6 ± 264
161–180	1.5	6	50	393.3 ± 25.9	249 ± 33	21 ± 4.3	11.4 ± 0.9	7.9 ± 0.4	27516.6 ± 104	8420.6 ± 91.8

**Fig. 1:**
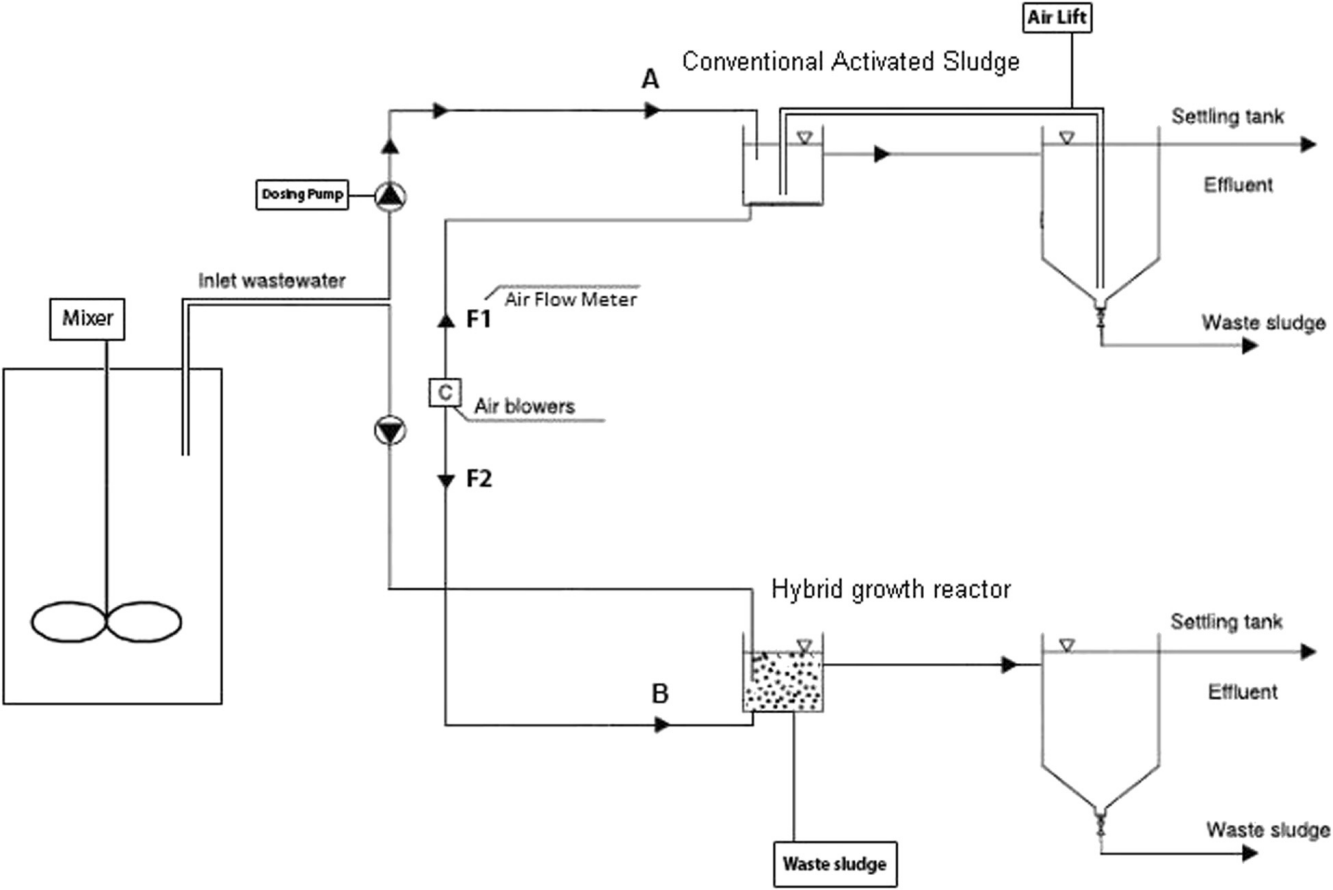
schematic pilot plant

All the materials used in this study was provided from Merck® Co. pH was measured by Elemetron pH-meter CP-411. TDS, Electrical Conductivity (EC), Chloride ion concentration were determined by HM digital Company’s products. All of measurements have been carried out according to DIN standard test methods. In this study, the pilot included two sections: control (conventional activated sludge system) and hybrid system. The hybrid system consists of fluidized beds and contains three phases as bed, biological film and influent wastewater which contains BOD_5_ (Biochemical Oxygen Demand), COD, TP (total phosphorous), and N-NH_4_^+^. The mentioned parameters removal by the biological film has been studied in this paper, according to the capability of biological film to oxidize and remove these items from wastewater [26]. Each of these sections has a biological reactor with an effective volume of 33.3 L and a settling tank with a volume of 19 L. Sludge returning was done only in conventional activated sludge reactor by air lift method. In the both reactors, aeration was done by the diffusers devised onto the floor and two flow-meters controlled the input air flow rate. The raw wastewater was maintained in a 300-L stock tank equipped with a stirrer. This wastewater was injected through two dosing pumps to the reactors. The principal section of the pilot (case section) had a hybrid growth system, where a polyethylene media with an active area of 500 m^2^/m^3^ and density of 95 kg/m^3^ was used. The reactors under investigation were made of Plexiglas sheets (Fig. 1).

To conduct this study, domestic wastewater samples were collected from Shahid Beheshti wastewater treatment plant, Tehran, Iran. This plant was established in 2002 with the flow rate of 150 m^3^/h. The treatment process in this plant is activated sludge with extended aeration. At the outset of the operation, in order to achieve stability conditions, the reactors were operationalized continuously for 10 days, with the COD being evaluated. After this period when COD removal percentage went beyond 80 % and the desired biofilm was formed in the filter media, investigation of other parameters started. Other parameters including BOD_5_, NH4^+^, Total Phosphorous (TP), Mixed Liquor Suspended Solids (MLSS), EC, and chloride were also measured. To examine the effect of sludge age, the return sludge value was employed. In order to return the sludge, airlift system was used. Since the hybrid growth process would result in further retention of bacteria in the reactor, thus sludge returning was applied only to the conventional reactor, where the hybrid reactor did not require sludge returning process, since it had a high MLSS concentration. The sludge return percentage was considered to be 25 % as the basis, followed by consideration of 50 and 75 % for sludge return. To study the effect of hydraulic retention time (HRT) on contaminants removal, after reaching stable conditions in the system, 2, 4, and 6 h HRTs were taken into account. Based on previous studies, the tolerance threshold of bacteria to the soluble salts in conventional activated sludge systems is 10,000 mg/L (1 %). In the present study, to examine the effect of soluble salts concentrations, the upper and lower limits of the mentioned threshold were considered. Since variations in the salt concentration in the reactor highly influence the growth of bacteria, thus, these changes of concentration from 0.5 to 1.5 % were applied very gradually. COD and TP tests were conducted by spectrophotometer, BOD_5_ by manometer, and the rest of experiments were carried out according to standard methods for the examination of water and wastewater. Every experiment was replicated three times to fulfill validity and accuracy.

To analyze the data, SPSS software (version 19) was used, where to compare the means, analysis of variance test was used considering a confidence level of 95 %.

## Results and discussion

### Variations in COD concentration during the operation of hybrid and conventional reactors for saline wastewater treatment

**Fig. 2:**
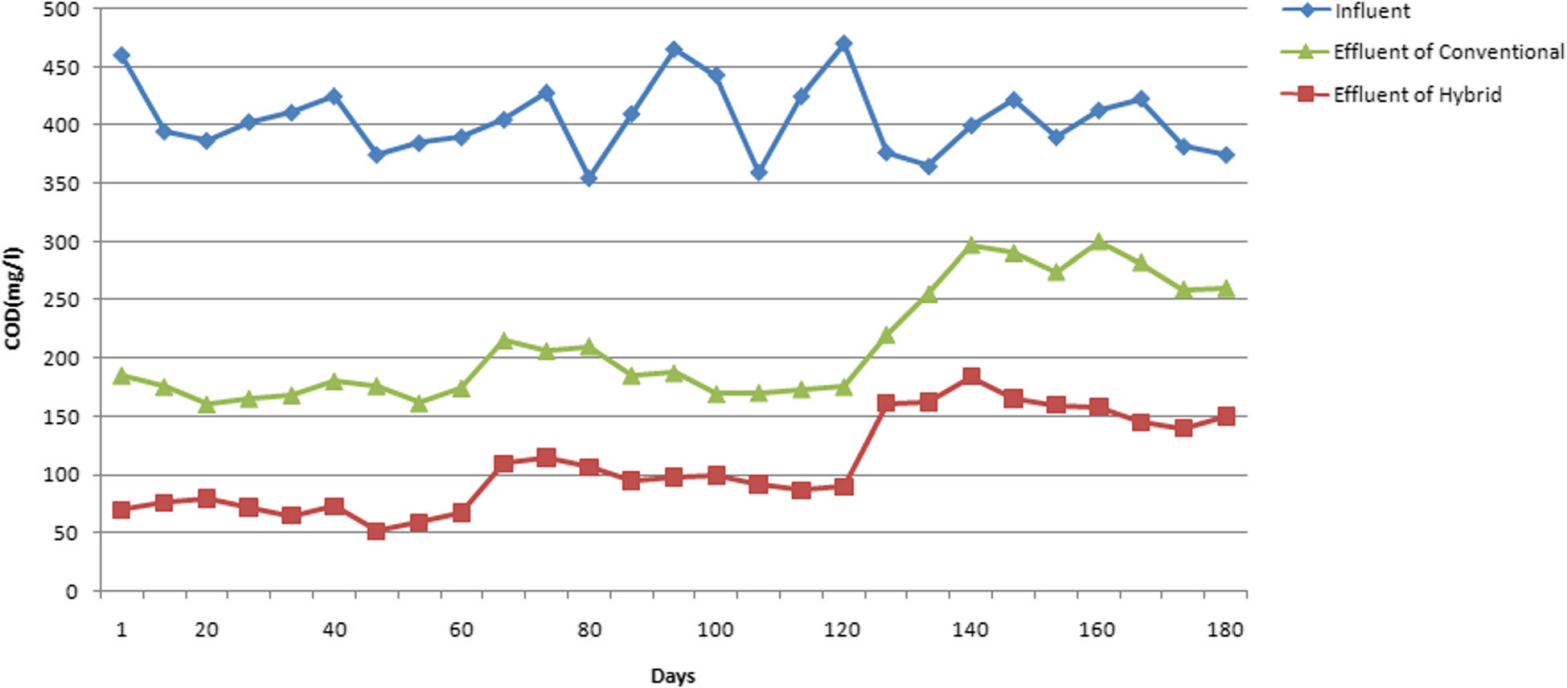
Variations of COD concentration during the operation of hybrid and conventional reactors

The results related to variations of COD concentration during the process in the hybrid and the conventional systems are indicated in Fig. 2. The average value of COD concentration in influent of both reactor’s has been decreased from 414 ± 40 mg/L in influent to 134.8 ± 52 and 219.1 ± 68.6 in hybrid and the conventional system, respectively. Szilagyi et al. [25] studied the treatment of household wastewater using a hybrid growth system (similar to the hybrid system of the current study), reporting removal percentage of 92 % at the influent concentration of 750 mg/L for COD. The obtained results indicated that the flexibility and resistance of this system at various loading rates, is relatively high. They also found that in some cases; this technology has a better performance in comparison with biofilm-based technologies and can be a suitable alternative to them. Nabi et al. [17] studied petrochemical wastewater using the combination of activated sludge process and polyurethane media with a specific area of 200 m^2^/m^3^. The results indicated that COD and ammonium nitrogen removal rates, would be significantly increased by using biofilm. This hybrid system managed to reduce the COD and ammonium nitrogen removal levels below the allowable amounts of wastewater discharge standards to different receptive sources and thus can be utilized as an efficient and reliable method of the treatment of petrochemical wastewater. Also, Qiqi et al. [19] indicated in their research that hybrid growth technology is an alternative and successful method for treatment of various wastewaters under different circumstances [19]. Cortes-Lorenzo et al. [7] obtained similar results by using submerged fixed bed biofilm reactor in wastewater with NaCl (3.7,24.1and44.1 g/L) and obtained these results by removing COD 89, 67.8, 39.5 %, respectively [7].

### The effect of salt concentration on COD removal

In order to study the relationship between salinity and COD removal, the performance of the reactors has been examined in different amounts of salt concentrations. At the salt concentration of 0.5 %, the mean value of input COD into two reactors was 403.4 ± 25.9 mg/l, while the value of COD of the hybrid and the conventional systems effluent were 84 ± 10.6 mg/l and 149.4 ± 46.3 mg/l, respectively. The considerably low amount of *p*-value (*p* = 0.001), resulted from the statistical analysis indicates that the difference between the output data of two reactors are significant at these conditions. The mean value of the input COD was about 417.8 ± 40.7 mg/L, when the salt concentration was about to 1 %. Also, the results showed that COD value of hybrid reactor’s effluent was noticeably lower than the conventional system (117.6 ± 8.7 mg/L and 218.3 ± 36.2 mg/L for the hybrid and conventional reactors, respectively). The results related to the statistical analysis also proved this results (*p* < 0.05).

Not only, increasing the salinity to higher values than 1 %, the efficiency of the reactors decreased noticeably, but also the COD removal behavior of the hybrid system is better than the conventional system. At the salt concentration of 1.5 %, the values of COD have been changed from 394.1 ± 21.4 mg/L in influent, to 203 ± 12.5 mg/l and 289.5 ± 28.2 mg/l, for the hybrid and conventional reactors effluents, respectively. The results of the statistical analysis also confirmed these observations (*p* < 0.05). Figure 2 demonstrates the relation of salt concentration values versus COD removal efficiency of the reactor. As shown in Fig. 2, when the salt concentration increases; COD removal efficiency in the hybrid reactor decreases from 80 % at the salt concentration of 0.5 to 48.5 % at the salt concentration of 1.5 %. Also, in the conventional system, the percentage of COD removal efficiency showed a decrease from 62.9 % in the salt concentration of 0.5 to 26.5 % at the salt concentration of 1.5 %.

**Fig. 3:**
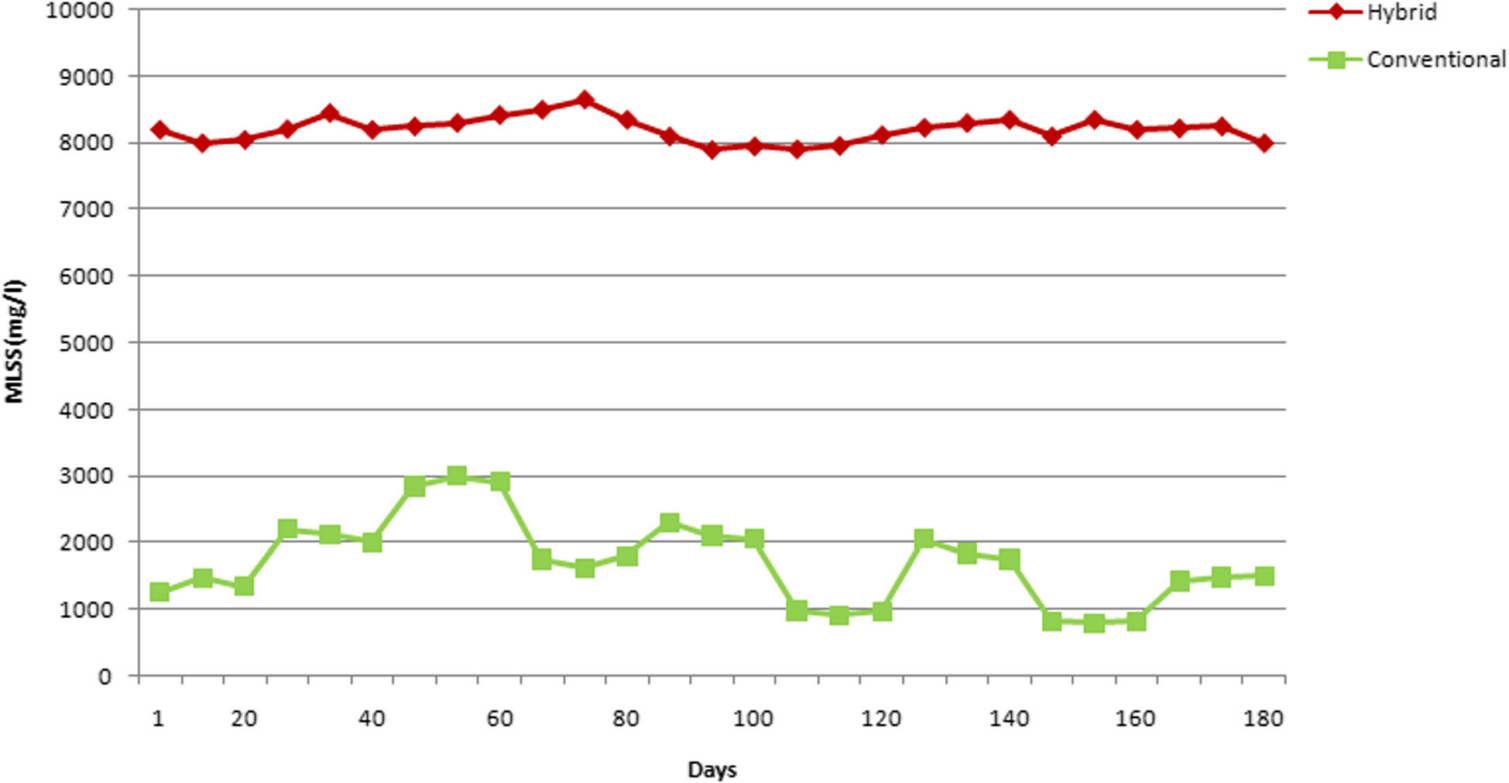
The diagram of variations in MLSS concentration during the operation

According to the results, it seems that the variation of COD removal efficiency values by changing the salinity of the conventional system, is related to MLSS concentrations. In the mentioned system, the value of MLSS concentration has been measured about 2125 ± 682 mg/L, 1608.3 ± 532.5 mg/L, and finally 1384.4 ± 475.6 mg/L at the salt concentrations of 0.5, 1 and 1.5 %, respectively. But in the hybrid system, the concentration of MLSS has remained approximately constant during the operation which was far greater than the conventional system (according to Fig. 3). The higher concentrations of MLSS in the hybrid system means higher sustainability and adaptability of the microorganisms in the systems. This higher MLSS concentration means the higher age of the sludge in the system which removes the demand for returning of sludge into the system in hybrid reactor, as system’s great advantage. More values for sludge age, modifies the COD removal efficiency in the system [21, 27]. Therefore, the observed better COD removal efficiency of the hybrid than the conventional system is reasonable.

### HRT for COD removal

HRT is a key operational parameter in biological systems. It inhibits washing out of the activated sludge mass, provides adequate contact time between the activated sludge and the treated organic substance, and always established a suitable concentration of biomass inside biological reactors [11]. The results of this study indicate that the COD values of both reactors input have been changed from 396.8 ± 32.2 mg/L at the HRT 2 h, to 144.6 ± 54.9 mg/Land 247.2 ± 37.2 mg/L, for hybrid and conventional systems, respectively. The results related to the statistical analysis also revealed that there is a significant difference between the output data of the hybrid and the conventional reactors at the HRT of 2 h (*p* < 0.05). By increasing the HRT of to 4 h, the COD value of hybrid and conventional reactors changed from 420.2 ± 22.3 mg/l in influent to 135.5 ± 51.7 mg/L and 219 ± 76.4 mg/L, in effluent, respectively. The results of the statistical analysis proved these observations (*p* < 0.05). At the hydraulic retention time of 6 h, the COD value of the reactors decreased from 398.3 ± 34.5 mg/L to 124.4 ± 53.6 mg/L and 191.1 ± 79.3 mg/L for the hybrid and the conventional systems, respectively. These observations have been confirmed by the statistical analysis tests (*p* < 0.05). According to the results, by extending the hydraulic retention time, the COD removal efficiencies of the hybrid and conventional reactors have been increases from 63 to 37 % at the retention time of 2 h, to 70 and 52 % at retention time of 6 h, respectively.

### Changes in BOD_5_ during the operation of hybrid and conventional reactors for treatment saline wastewater treatment

**Fig. 4:**
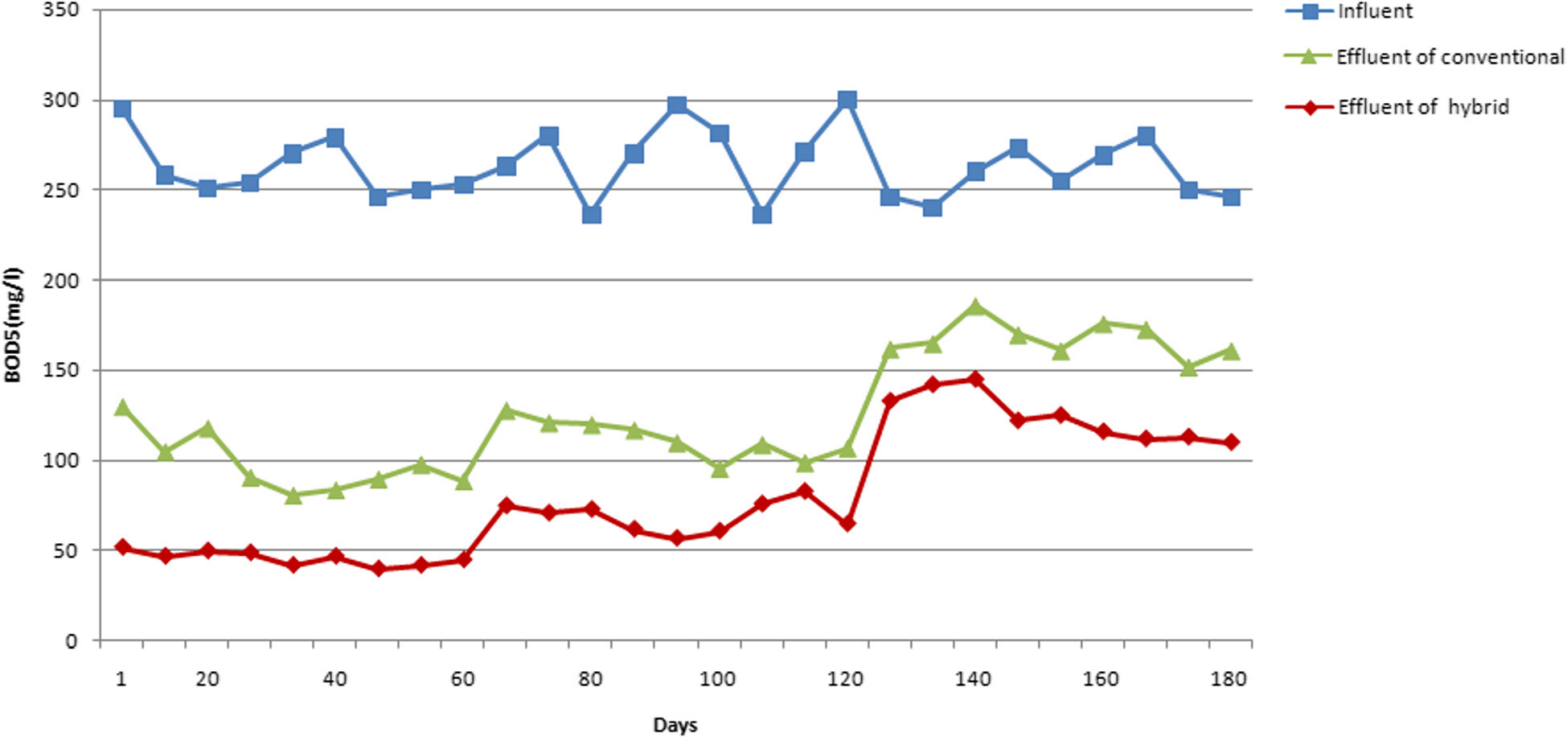
Variations of BOD_5_ concentration during the operation of hybrid and conventional reactors

The results related to variations in the BOD_5_ concentration during the operation of hybrid and conventional activated sludge reactors are shown in Fig. 4. The values of BOD_5_ have been changed from 250.5.3 ± 24.5 mg/L in influent to 102.5.4 ± 35.2 mg/L in the hybrid system and to 121.3 ± 29.7 mg/L in the conventional system.

### The effect of salt concentration on BOD_5_ removal rate

Biswas et al. (2013) removed BOD_5_ about to 97 % using MBBR (Moving Biological Bed Reactor) system which is quite similar to our hybrid reactor [5]. In order to study the effect of salinity on BOD_5_ removal, the performance of the reactors has been studied in different amounts of salt concentrations. At the salt concentration of 0.5 %, the BOD_5_ value of two reactors influent decreased from 245.3 ± 12.3 mg/L to 67.3 ± 8.3 mg/L and 94.5 ± 11.3 mg/L in the effluent of the hybrid and the conventional systems, respectively. In other words, BOD_5_ removal at the salt concentration of 0.5 % was 72.5 % for the hybrid, and 61.4 % for the conventional system. The results related to the statistical analysis also revealed that there is a significant difference between the output data of the hybrid system and the conventional reactor at the salt concentration of 0.5 % (*p* < 0.05). Increasing salinity affects the BOD_5_ removal performance of the reactors, negatively. The BOD_5_ value of influent of both reactors changed from 245.5 ± 25.9 mg/L to 94.2 ± 8.4 mg/L and 111.8 ± 10.5 mg/L for hybrid and conventional reactors, respectively, at the salt concentration of 1 %. The results related to the statistical analysis confirmed these observations (*p* < 0.05). By increasing the salt concentration to 1.5 %, the BOD_5_ value in influent of both reactors decreased from 260.8 ± 30.9 mg/L to 146.1 ± 17.3 mg/L and 157.6 ± 15.5 mg/L, in hybrid and conventional reactors effluents, respectively. The results related to the statistical analysis confirmed these results, too (*p* < 0.05).

### The effect of retention time on BOD_5_ removal rate

At the hydraulic retention time of 2 h, the BOD_5_ values of both reactors have been changed from 250 ± 27 mg/L to 112.5 ± 40.7 mg/L for hybrid and 130.1 ± 27.7 mg/L, for conventional systems effluent. At the hydraulic retention time of 4 h, the input BOD_5_ value of both reactors influent was determined 256.7 ± 24.5 mg/L, while the value of this parameter of the hybrid and conventional reactors was measured 100.2 ± 35.1 mg/L and 117.7 ± 34.8 mg/L, respectively. At the hydraulic retention time of 6 h, BOD_5_ values of reactors influent have been changed from 244.2 ± 23.2 mg/L to 94.8 ± 31 mg/L and 116.2 ± 27.5 mg/L in hybrid and conventional reactors effluents, respectively. The results of the statistical analysis also proved these observations (*p* < 0.05).

### Variations of NH_4_^+^ concentration during the operation of the hybrid and the conventional reactors to saline wastewater treatment

**Fig. 5:**
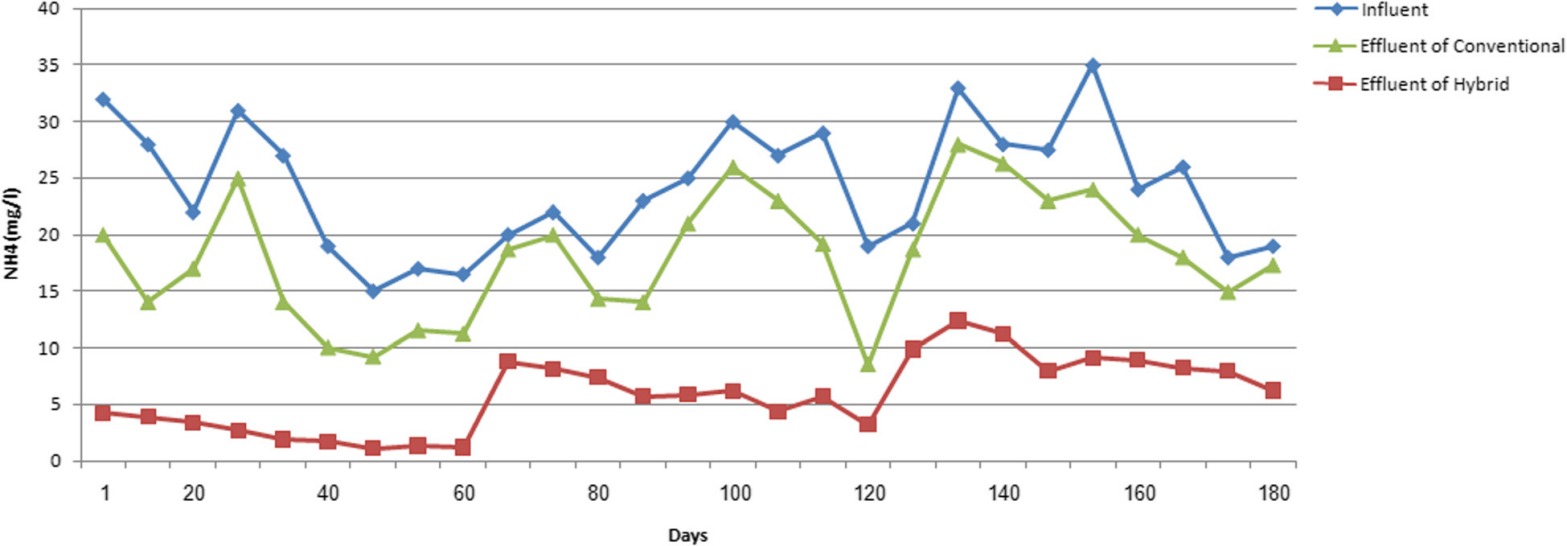
Variations in NH_4_^+^ concentration during the operation of the hybrid and conventional reactors

The results related to variations in the NH_4_^+^ concentration in the aeration reactors with the hybrid and the conventional activated sludge system are shown in Fig. 5. According to Fig. 5, the average concentration of NH_4_^+^ of the influent have been decreased from 24.1 ± 5.5 mg/L to 5.8 ± 3.2 mg/L for hybrid and 19.7 ± 5.4 mg/L for the conventional reactors. Based on the results, the nitrification in the hybrid and the conventional reactors were about to 76 and 18 %, respectively. According to literature, in the hybrid system, the rates of organic substances removal and nitrification processes are dependent on dissolved oxygen concentration. Since, by inhibiting the outflow of oxygen, the media provides optimal conditions for ammonia removal, thus, the efficiency of this process is far greater in the hybrid system. The level of dissolved oxygen for full nitrification in the hybrid system has been proposed to be between 3 and 4 mg/L [13].

### The effect of salt concentration on NH_4_^+^ removal

According to the results, at the salt concentration of 0.5 %, the mean concentration of NH_4_^+^ has been decreased from 23 ± 6.5 mg/L to 2.3 ± 1.2 mg/L in hybrid and 17.9 ± 6.5 mg/L in conventional system reactor. The mean concentration of NH_4_^+^ in influents of two reactors, has been changed from 23.6 ± 4.3 mg/L to 6.1 ± 1.7 mg/L and 19.1 ± 3.9 mg/L for hybrid and conventional systems effluents, respectively, by increasing the salt concentration to 1 %. At the salt concentration of 1.5 %, the mean concentration of NH_4_^+^ in influent of both reactors showed a decrease from 25.7 ± 5.8 mg/L to 9 ± 1.8 mg/L in the hybrid and 22.1 ± 5 mg/L in the conventional system. The results related to the statistical analysis proved that there is a significant difference between the output data in both reactors in various salt concentrations (*p* < 0.05).

These results are compatible with the reports in similar conditions in literature. For instance, Bassin et al. [4] analyzed NH_4_^+^ removal from domestic salt wastewater with 8000 mg/L concentration ion-Cl^−^ using a special kind of MBBR system, and could remove 90 % of ammonia within 160 days [4]. Also, Pal et al. [18] could decrease ammonia from 10 to 2 mg/L using another MBBR system [18].

According to the results obtained from experiments, ammonia removal values were observed in 90, 74, and 65 % in the hybrid and 22, 19, 14 % in the conventional system when the salt concentrations were 0.5, 1 and 1.5 %, respectively. Also, in all conditions the hybrid reactors performance was remarkably better than the conventional system. It seems that, the reason for this significantly better ammonia removal performance of the hybrid system is due to the relatively higher concentration of dissolved oxygen than conventional reactor, which were observed 2.96 mg/L in the hybrid and 0.51 mg/L in the conventional reactor, during their performance, even if in the same flow rate of input air into both of them (0.73 L/min).

### The effect of retention time on NH_4_^+^ removal rate

The ammonia removal behavior of both systems has been investigated in various HRTs. At the HRT about to 2 h, the mean concentration of NH_4_^+^ has been changed from 24.8 ± 5.4 mg/L in influent of both reactors to 7.6 ± 3.2 mg/L and 21.4 ± 4.7 mg/L in the hybrid and the conventional system’s effluents, respectively. By increasing the HRT to 4 h, the mean concentration of NH_4_^+^ in influent of both reactors decreased from 26.8 ± 4.7 mg/L to 5.5 ± 2.8 mg/L and 21.7 ± 5.3 mg/L, in the hybrid and the conventional system effluents, respectively. At the HRT of 6 h, the mean concentration of NH_4_^+^ in influent of both reactors showed a reduction from 20.7 ± 5.1 mg/L to 4.3 ± 2.8 mg/l in the hybrid and 16 ± 4.6 mg/L the conventional systems effluents. Also, the results related to the statistical analysis revealed that there is a significant difference between the output data of the hybrid and the conventional reactors in various HRTs (*p* < 0.05).

### Variations in phosphorus concentration during the operation of hybrid and conventional reactors for saline wastewater treatment

**Fig. 6:**
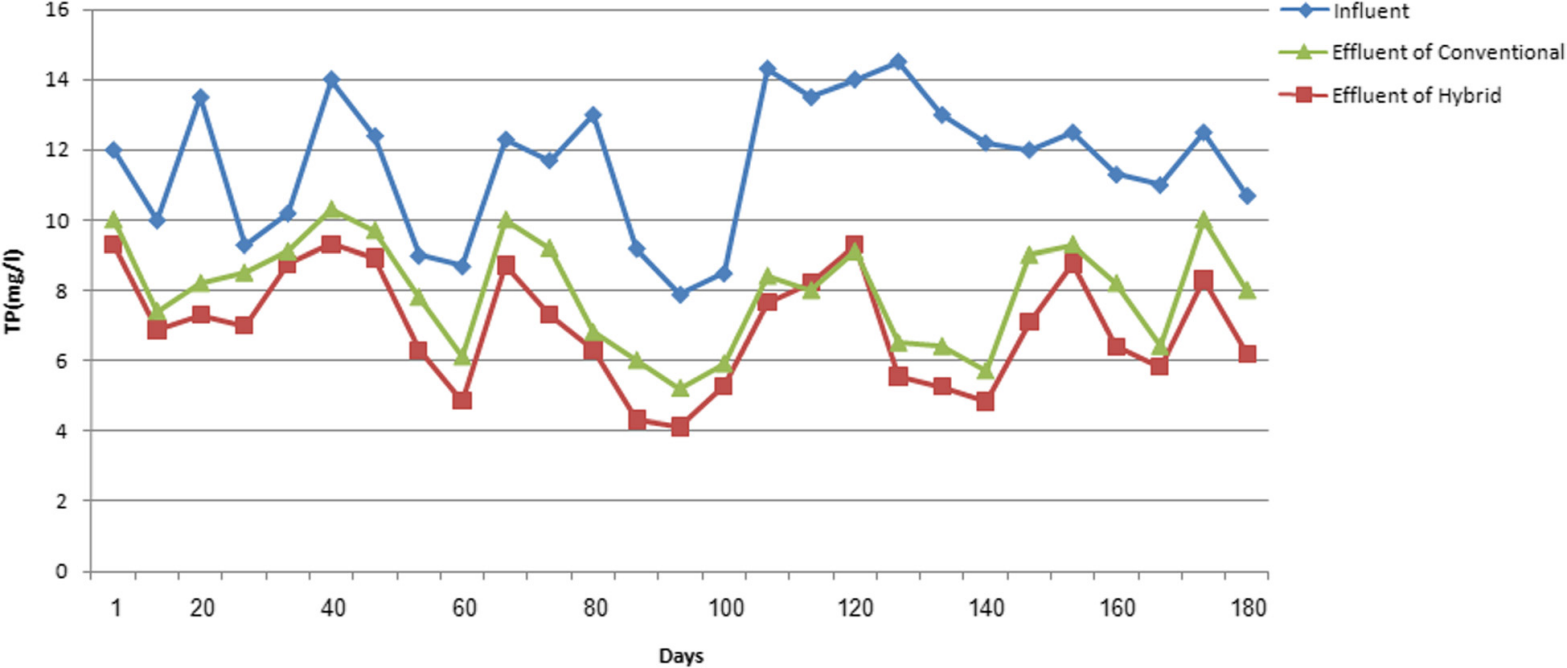
Variations of phosphorus concentration during the operation of the hybrid and conventional reactors

The results related to variations in the phosphorus concentrations during the operation conditions in both systems has been shown in Fig. 6.

According to Fig. 6, the mean concentration of phosphorus has been reduced from 11.6 ± 1.9 mg/L in the influent of both systems, to 6.9 ± 1.6 mg/L and 7.9 ± 1.5 mg/L in effluents of the hybrid and the conventional systems, respectively.

### The effect of salt concentration on TP removal

Various studies have been carried out on TP removal from wastewater by different researchers. Azizi et al. [3] could remove 40.2 % of total phosphorous from wastewater by using MBBR system. At the salt concentration of 0.5 %, the mean concentration of TP in both reactors influents was changed from 11 ± 2 mg/L to 7.6 ± 1.5 and 8.5 ± 1.3, in the hybrid and the conventional systems, respectively. Also, at the salt concentration of 1 %, TP mean concentration was decreased from 11.6 ± 2.4 mg/L to 6.8 ± 1.8 mg/L in the hybrid and 7.6 ± 1.7 mg/L in the conventional reactors. By increasing the salt concentration to 1.5 %, the mean concentration TP was reduced from 12.1 ± 1.1 mg/L to 6.4 ± 1.3 mg/L and 7.7 ± 1.5 mg/L, in the hybrid and the conventional systems, respectively. The results of the statistical analysis proved the significant difference between the output data of the hybrid and the conventional reactors at the various salt concentration (*p* < 0.05).

### The effect of retention time on TP removal

At the HRT of 2 h, the mean concentration of TP in the input of both reactors has been changed from 12.4 ± 1.2 mg/L to 6.8 ± 1.5 mg/L in the hybrid and 7.8 ± 1.6 mg/L in the conventional systems, respectively. By increasing the HRT to 4 h, the mean concentration of TP was decreased from 10.5 ± 2 mg/L to 6.7 ± 1.9 mg/l and 7.9 ± 1.7 mg/l, in the effluent of the hybrid and the conventional reactors respectively. Also, at HRT about to 6 h, the mean concentration of TP has been reduced from 11.7 ± 2 mg/L to 7.2 ± 1.5 mg/L in the hybrid and 8.1 ± 1.3 mg/L in the conventional systems. The statistical analysis results for experiments in different HRTs, revealed the significant difference between the output data in the hybrid and the conventional reactors (*p* < 0.05), too.

### Effect of the sludge recycling ratio on mixed liquor suspended solids (MLSS) concentration in the conventional reactor

The effect of returned-sludge ratio has been evaluated for the conventional system. When the returned-sludge ratio of 25 % (on the basis of the reactor’s input), the MLSS concentration has been measured about to 1037.2 ± 252.35 mg/L, in the conventional reactor. By increasing this ratio to 50 %, the concentration of MLSS was about to 1765.5 ± 287.9 mg/L, in this reactor. Finally, in 75 % returned-sludge ratio, the MLSS concentration increased to 2315.5 ± 478.3 mg/L.

In the hybrid reactor, according to this fact that the concentration of MLSS was noticeably high (8100 ± 155 mg/L), there was no need to insert returned-sludge into the reactor.

## Conclusions

In this study, the removal performance of the conventional activated sludge system and a fluidized bed positioned in a biological reactor of an activated sludge process (as the novel modified reactor, named as the hybrid system) have been compared with each other with the aim of saline municipal wastewater treatment. The removal of COD, BOD_5_, NH_4_^+^, and TP have been evaluated in various salinities and HRTs (0.5, 1 and 1.5 % of salt concentrations and 2, 4 and 6 h). The results showed that the removal performances of hybrid system in all conditions were noticeably better than the conventional system. In addition, the hybrid system does not need to return sludge, due to its approximately constant concentration of MLSS, as its great advantage. Dissolved oxygen concentration of the hybrid system was observed relatively higher, in comparison with the conventional system, as it’s another advantage.

These features improve economic indices in wastewater treatment plants concerning the costs required for aeration and sludge return facilities. According to the results, it is concluded that the novel hybrid system it strongly suitable to be used in the treatment of saline municipal wastewaters in various due to its potencies and advantages.

## Abbreviations

BOD: Biochemical Oxygen Demand
COD: Chemical Oxygen Demand
EC: Electrical Conductivity
HRT: Hydraulic retention rime
MBBR: Moving Biological Bed Reactor
MLSS: Mixed Liquor Suspended Solids
TDS: Total Dissolved Solid
TP: Total phosphorous

## References

[1] Amin MM, Khiadani MH, Fatehizadeh A, Taheri E (2014). Validation of linear and non-linear kinetic modeling of saline wastewater treatment by sequencing batch reactor with adapted and non-adapted consortiums. Desalination.

[2] Angelakis AN, Gikas P (2014). Water reuse: overview of current practices and trends in the world with emphasis in EU states. Water Util J.

[3] Azizi S, Valipour A, Sithebe T (2013). Evaluation of different wastewater treatment processes and development of a modified attached growth bioreactor as a decentralized approach for small communities. Sci World J.

[4] Bassin JP, Kleerebezem R, Muyzer G, Rosado AS, van Loosdrecht MC, Dezotti M (2012). Effect of different salt adaptation strategies on the microbial diversity, activity, and settling of nitrifying sludge in sequencing batch reactors. Appl Microbiol Biotechnol.

[5] Biswas K, Taylor MW, Turner SJ (2014). Successional development of biofilms in moving bed biofilm reactor (MBBR) systems treating municipal wastewater. Appl Microbiol Biotechnol.

[6] Chai H-X, Chen W, He Q, Zhou J (2015). Effects of volumetric load in an anaerobic sequencing batch biofilm treating industrial saline wastewater. Environ Technol.

[7] Cortes-Lorenzo C, Rodriguez-Diaz M, Lopez-Lopez C, Sanchez-Peinado M, Rodelas B, Gonzalez-Lopez J (2012). Effect of salinity on enzymatic activities in a submerged fixed bed biofilm reactor for municipal sewage treatment. Bioresour Technol.

[8] Cortés-Lorenzo C, Sipkema D, Rodríguez-Díaz M, Fuentes S, Juárez-Jiménez B, Rodelas B, Smidt H, González-López J (2014). Microbial community dynamics in a submerged fixed bed bioreactor during biological treatment of saline urban wastewater. Ecol Eng.

[9] Di Bella G, Giustra M, Freni G (2014). Optimisation of coagulation/flocculation for pre-treatment of high strength and saline wastewater: Performance analysis with different coagulant doses. Chem Eng J.

[10] Duan J, Fang H, Su B, Chen J, Lin J (2015). Characterization of a halophilic heterotrophic nitrification–aerobic denitrification bacterium and its application on treatment of saline wastewater. Bioresour Technol.

[11] Farzadkia M, Kalantari RR, Mousavi SG, Jorfi S, Gholami M (2010). Treatment of Synthetic Wastewater Containing Propylene Glycol by a Lab Scale Fixed Bed Activated Sludge Reactor. Water Wastewater J.

[12] Gupta VK, Ali I, Saleh TA, Nayak A, Agarwal S (2012). Chemical treatment technologies for waste-water recycling—an overview. RSC Adv.

[13] Hansler S (2008). Conceptual Level Design for MBBR Option. Excellence in Environmental Consulting Services.

[14] Jang D, Hwang Y, Shin H, Lee W (2013). Effects of salinity on the characteristics of biomass and membrane fouling in membrane bioreactors. Bioresour Technol.

[15] Mannina G, Cosenza A, Di Trapani D, Capodici M, Viviani G (2016). Membrane bioreactors for treatment of saline wastewater contaminated by hydrocarbons (diesel fuel): An experimental pilot plant case study. Chem Eng J.

[16] Mseddi S, Chakchouk I, Aloui F, Sayadi S, Kallel M (2014). Development of a process for the treatment of fish processing saline wastewater. Desalin Water Treat.

[17] Nabi BG, Jafari SB, Vosoogh A, Baghvand A, Daryabeigi ZA (2012). Efficiency Of Active Sludge Process Treatment Of Petrochemical Industries Wastewater By Using Of Biofilm (Case Study: Imam Khomeiny Port Petrochemical Complex).

[18] Pal L, Kraigher B, Brajer-Humar B, Levstek M, Mandic-Mulec I (2012). Total bacterial and ammonia-oxidizer community structure in moving bed biofilm reactors treating municipal wastewater and inorganic synthetic wastewater. Bioresour Technol.

[19] Qiqi Y, Qiang H, Ibrahim HT (2012). Review on moving bed biofilm processes. Pak J Nutr.

[20] Qiu G, Ting Y-P (2013). Osmotic membrane bioreactor for wastewater treatment and the effect of salt accumulation on system performance and microbial community dynamics. Bioresour Technol.

[21] Rene ER, Kim SJ, Park HS (2008). Effect of COD/N ratio and salinity on the performance of sequencing batch reactors. Bioresour Technol.

[22] Roy D, Rahni M, Pierre P, Yargeau V (2016). Forward osmosis for the concentration and reuse of process saline wastewater. Chem Eng J.

[23] Selvam S, Manimaran G, Sivasubramanian P, Balasubramanian N, Seshunarayana T (2014). GIS-based evaluation of water quality index of groundwater resources around Tuticorin coastal city, South India. Environ Earth Sci.

[24] Shen Q-H, Gong Y-P, Fang W-Z, Bi Z-C, Cheng L-H, Xu X-H, Chen H-L (2015). Saline wastewater treatment by Chlorella vulgaris with simultaneous algal lipid accumulation triggered by nitrate deficiency. Bioresour Technol.

[25] Szilagyi N, Kovacs R, Kenyeres I, Csikor Z (2011). Performance of newly developed bio film–based wastewater treatment technology. 1 st IWA Central Asian Regional Young water professional conference.

[26] Tchobanoglous G, Burton FL, Stensel HD (2003). Wastewater engineering: treatment and reuse.

[27] Uygur A, Kargı F (2004). Salt inhibition on biological nutrient removal from saline wastewater in a sequencing batch reactor. Enzym Microb Technol.

[28] Wang R, Zheng P, Ding A-q, Zhang M, Ghulam A, Yang C, Zhao H-P (2016). Effects of inorganic salts on denitrifying granular sludge: The acute toxicity and working mechanisms. Bioresour Technol.

[29] Warner NR, Christie CA, Jackson RB, Vengosh A (2013). Impacts of shale gas wastewater disposal on water quality in western Pennsylvania. Environ Sci Technol.

[30] Winkler M-K, Bassin J, Kleerebezem R, Van der Lans R, van Loosdrecht M (2012). Temperature and salt effects on settling velocity in granular sludge technology. Water Res.

[31] Yuan R, Wang Z, Hu Y, Wang B, Gao S (2014). Probing the radical chemistry in UV/persulfate-based saline wastewater treatment: Kinetics modeling and byproducts identification. Chemosphere.

[32] Zhang L, Zhu K, Li A (2016). Differentiated effects of osmoprotectants on anaerobic syntrophic microbial populations at saline conditions and its engineering aspects. Chem Eng J.

